# Data-driven strategies for immunoradiotherapy in uveal melanoma: the role of artificial intelligence

**DOI:** 10.3389/fphar.2026.1762154

**Published:** 2026-01-29

**Authors:** Dongling Gu, Yi Feng, Hongyan Li

**Affiliations:** 1 Shantou University, Shantou, China; 2 Department of Anesthesiology, The Affiliated Traditional Chinese Medicine Hospital, Southwest Medical University, Luzhou, Sichuan, China; 3 Luzhou Key Laboratory of Research for Integrative on Pain and Perioperative Organ Protection, Luzhou, Sichuan, China

**Keywords:** artificial intelligence, immunoradiotherapy, personalized medicine, tumor immune microenvironment, uveal melanoma

## Abstract

Uveal melanoma (UM) represents the most common primary intraocular malignancy in adults and remains a formidable clinical challenge due to its high metastatic potential and characteristically limited response to conventional systemic therapies. While the combination of radiotherapy and immunotherapy has emerged as a promising multimodal strategy for managing this complex malignancy, its efficacy is significantly constrained by profound individual variations in tumor biology, immune microenvironment composition, and dynamic treatment response patterns. In recent years, artificial intelligence (AI) has fundamentally transformed the landscape of precision oncology by enabling sophisticated image analysis, robust data-driven prediction, and seamless integration of heterogeneous multi-omics information. Within the specific context of uveal melanoma, AI-driven computational models have demonstrated significant potential to accurately predict therapeutic outcomes, quantitatively characterize the tumor immune microenvironment, and optimize radiotherapeutic strategies on a personalized basis. This comprehensive review critically examines and synthesizes recent progress in AI applications for immunoradiotherapy in uveal melanoma, systematically exploring their transformative potential to refine diagnostic accuracy, enhance treatment precision, and ultimately improve long-term patient outcomes through intelligent, data-driven personalized medicine approaches that bridge multiple disciplinary boundaries.

## 1 Introduction

Uveal melanoma (UM) arises from melanocytes of the uveal tract, most frequently within the choroid (about 85%), followed by the ciliary body and iris (Demirci et al., 2023; Spagnolo et al., 2012). Its biological and clinical features differ markedly from cutaneous melanoma. The annual incidence in Europe and the United States is 5–7 cases per million, and choroidal melanoma accounts for roughly 85%–90% of all UM cases (Sorrentino et al., 2024). Current local therapies such as enucleation, plaque brachytherapy and proton beam radiation have substantially improved primary tumour control, yet they have not prevented the development of distant metastases, which remain the principal cause of mortality (Kulbay et al., 2024). Systemic immunotherapies that have reshaped treatment outcomes in many solid tumours have yielded only limited benefits in UM. This shortfall reflects the tumour’s low mutational burden, distinctive antigenic landscape and the immune-privileged nature of the eye (Fu et al., 2022).

Radiotherapy occupies a pivotal position in UM management (Yilmaz et al., 2024). Beyond its direct cytotoxic effect and capacity for precise local ablation, radiation modulates the tumour microenvironment in ways that may enhance immune recognition. Ionizing radiation can induce immunogenic cell death, increase antigen presentation and promote T-cell infiltration through diverse molecular pathways (Jiang et al., 2024; Xu et al., 2025). The magnitude and persistence of these immune effects vary significantly between patients, influenced by radiation dose, fractionation strategies and tumour genotype. Recent analyses of emerging radiotherapy modalities in UM underscore both established approaches and new technological developments (Semeniuk et al., 2024). Immunoradiotherapy, achieved by combining radiotherapy with immune checkpoint inhibitors or adoptive cellular strategies, aims to convert immunologically cold tumours into hot ones by amplifying both local and systemic immunity (Tang et al., 2023). The effectiveness of such combination treatments depends crucially on precise patient selection and adaptable treatment optimisation, both of which could be markedly strengthened by AI-based tools.

The incorporation of AI into oncology is transforming the ability to interpret complex tumour biology and personalise treatment (Nardone et al., 2024). In UM, AI can integrate multimodal datasets, including medical imaging, histopathology and comprehensive omics profiles, to identify latent patterns associated with immune activation and radiosensitivity (Wan et al., 2025). By linking radiomic and molecular features to clinical outcomes, AI enables the development of high-resolution predictive frameworks capable of guiding rational, precision immunoradiotherapy (Li et al., 2025).

Importantly, in the context of uveal melanoma, artificial intelligence should not be viewed as a generic analytical add-on, but rather as a unifying framework that enables true AI-driven immunoradiotherapy. Unlike conventional AI applications in oncology that primarily focus on diagnosis or outcome prediction in isolation, AI-driven immunoradiotherapy specifically addresses three interrelated challenges: identifying patients most likely to benefit from combined radiation–immune strategies, quantitatively modeling the dose–immunity interaction, and dynamically predicting treatment response to support adaptive therapeutic decision-making. By integrating radiotherapy parameters, immune landscape features, and multi-omics data into a coherent computational paradigm, AI provides a rational basis for transforming empirically combined therapies into biologically informed, precision-guided treatment strategies tailored to the unique immune-cold nature of uveal melanoma.

**FIGURE 1:**
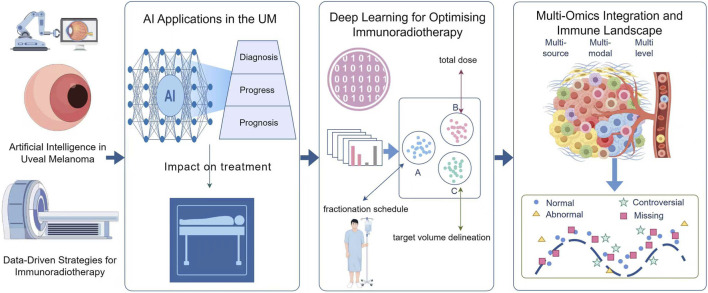
A framework for data-driven immunoradiotherapy strategies targeting uveal melanoma.

This review examines the intersection of AI, radiotherapy and immunotherapy in uveal melanoma, highlighting advances that are reshaping the field, ongoing barriers to clinical integration and future directions with potential to redefine therapeutic strategies for this challenging disease (Figure 1).

## 2 The application of AI in diagnosing and prognosing UM influences treatment decisions

Accurate diagnosis and reliable prognostic evaluation are essential for effective management of uveal melanoma. Conventional ophthalmic imaging techniques, including ultrasonography, optical coherence tomography (OCT) and fun dus photography, are routinely used to characterise tumours, while histopathology remains the definitive method for diagnostic confirmation (Kalemaki et al., 2020; Kaluzny et al., 2009). These approaches, however, depend heavily on clinician expertise and subjective visual interpretation, which may fail to capture subtle morphological cues with prognostic relevance. Deep learning approaches have shown exceptional capacity for automated image analysis and quantitative feature extraction, offering reproducible and scalable tools for comprehensive tumour assessment (Han et al., 2025). Dadzie and colleagues reported that convolutional neural networks (CNNs) trained on fundus photographs, OCT and ultrasonography consistently achieved diagnostic AUC values above 0.90, outperforming traditional handcrafted feature-based machine-learning models (Dadzie et al., 2025). In one study, a CNN differentiated uveal melanoma from choroidal nevi with more than 95% accuracy using high-resolution fundus imaging (Adusei et al., 2024). Integrating radiomic features derived from MRI with AI algorithms further enabled volumetric and texture-based analyses that correlated strongly with tumour aggressiveness and metastatic propensity (Theek et al., 2018).

Beyond diagnostic refinement, AI is increasingly applied to prognostic prediction, including metastatic risk stratification. Kelly et al. developed and validated a prognostic scoring system named MUMPS for patients with metastatic choroidal melanoma receiving immune checkpoint inhibitor therapy (Kelly et al., 2021). This score is based on three readily obtainable clinical variables: whether the time from initial diagnosis to metastasis exceeded 2 years, the presence of bone metastases, and serum lactate dehydrogenase levels below 1.5 times the upper limit of normal. Patients are categorized into favorable, intermediate, and poor prognosis groups based on the number of favorable factors they meet. Patients with a favorable MUMPS score may achieve longer survival with anti-PD-1/L1 monotherapy compared to combination immunotherapy.

AI-driven computational pathology represents another rapidly advancing direction. Deep learning applied to digitised whole-slide images can quantify immune-cell infiltration, analyse nuclear architecture and estimate proliferative activity with high reproducibility (Su et al., 2025a). When linked to comprehensive immune gene-expression signatures, these image-based biomarkers reliably identify immune-cold UM phenotypes, which are typically associated with limited responsiveness to immunotherapy (Messina et al., 2012; Vogele et al., 2022).

Additionally, AI has demonstrated significant value in longitudinal treatment monitoring and response assessment. Deep learning algorithms trained on serial imaging datasets can detect subtle morphological or vascular changes during treatment courses, allowing for real-time evaluation of therapeutic response dynamics (Esteva et al., 2019). By systematically tracking radiomic‐feature evolution over time, clinicians can identify early indicators of immune activation or emerging resistance patterns—thus enabling adaptive modifications to treatment strategy. In effect, this dynamic monitoring capability transforms disease management from a reactive approach to an anticipatory paradigm, aligning closely with the goals of precision oncology.

Importantly, AI-based diagnostic and prognostic assessments can directly inform immunoradiotherapy decisions in uveal melanoma. For instance, patients identified by AI models as having a high metastatic risk—based on integrated imaging and molecular features—may be considered for early combined radiotherapy and immunotherapy rather than radiotherapy alone. Similarly, AI-driven immune profiling can identify profoundly immune-cold tumors, supporting the use of radiotherapy regimens optimized for immune priming followed by appropriately timed immunotherapy. These scenarios illustrate how AI predictions can be translated into actionable, patient-specific immunoradiotherapy strategies.

## 3 Radiomics and deep learning for optimising immunoradiotherapy

From the perspective of immunoradiotherapy, the key value of AI lies not simply in optimizing radiation delivery, but in quantitatively modeling the nonlinear and patient-specific interactions between radiation dose, fractionation, and immune activation, which are otherwise difficult to capture using traditional radiobiological frameworks.

The fundamental rationale for combining radiotherapy and immunotherapy in UM lies in their potential synergistic interaction (Loi et al., 2017). Radiation not only induces direct DNA damage and cell death, but also releases tumour‐associated antigens and damage-associated molecular patterns (DAMPs), thereby facilitating enhanced immune-system recognition and engagement (Hoover et al., 2022). However, the immunologic consequences of radiation exposure are inherently dose‐dependent and context‐sensitive, creating complex optimisation challenges. Determining optimal dosing parameters—total dose, fractionation schedule, target volume delineation—requires sophisticated analytical approaches where AI can play a decisive role (Ibrahim et al., 2025). While AI-guided radiotherapy optimization and adaptive treatment strategies have been extensively validated in several non-uveal malignancies, direct clinical validation in uveal melanoma remains limited. Accordingly, such examples are discussed here as transferable methodological insights, rather than evidence directly established in UM.

AI-enabled radiomics offers quantitative characterisation of tumour heterogeneity, revealing microstructural features that modulate radiosensitivity and therapeutic response. Texture descriptors such as entropy, skewness and kurtosis derived from MRI or CT capture spatial patterns associated with hypoxia or necrosis, regions often resistant to radiation-induced killing (Foti et al., 2022). Deep-learning frameworks can integrate these radiomic variables with clinical and genomic data to model dose–response relationships with high fidelity (Li et al., 2024). Evidence from other malignancies, including head and neck and cervical cancers, shows that AI systems can estimate tumour-control probability and normal-tissue complication probability more accurately than conventional radiobiological models, and similar methodologies are now being adapted for UM (Tajiki et al., 2023; Liu et al., 2021; Tai et al., 2022).

Beyond static predictions, AI also enables dynamic, adaptive treatment strategies through continual learning. By analysing serial imaging during treatment, machine-learning models can detect evolving signatures of tumour regression, oedema or perfusion changes that reflect ongoing immune activation. These temporal signals allow AI algorithms to propose real-time adjustments to radiation intensity or to determine optimal intervals for immunotherapy. Recurrent neural networks, for example, have been used to forecast trajectories of immune-cell infiltration following irradiation, enabling synchronisation of checkpoint-inhibitor administration with periods of maximal immunogenicity (Liu et al., 2021).

Radiogenomic mapping represents another important frontier. By correlating radiomic patterns with transcriptomic or proteomic features, AI systems can infer molecular pathways that underlie heterogeneous treatment responses (Wu et al., 2018). In UM, specific radiomic signatures from T1-weighted and diffusion-weighted MRI have been linked to BAP1 loss and macrophage infiltration, both strong determinants of immune activation and metastatic risk (Figueiredo et al., 2020). Incorporating these multimodal features into predictive models may help clinicians estimate how effectively radiation can prime the tumour microenvironment for immunotherapy.

AI-guided radiotherapy planning further enhances treatment precision and safety. Deep-learning–based contouring improves the accuracy of target delineation while reducing radiation exposure to sensitive ocular structures such as the lens, retina and optic nerve (El Basha et al., 2018). Advanced dose-optimisation algorithms can generate highly individualised plans that balance tumour control with toxicity reduction (Parker et al., 2020). Together, these tools form the basis of an integrated, feedback-driven radiotherapy workflow—an essential prerequisite for implementing precision immunoradiotherapy in UM.

An illustrative recent application: a decision-support AI tool for treatment-modality selection in UM (stereotactic radiotherapy (SRT) vs. protons) achieved accuracy of 81%, 77%, 91% and 93% for different toxicity profiles (maculopathy/optic neuropathy; neovascular glaucoma; retinopathy; dry-eye syndrome) based on clinical characteristics (n = 66) [AI vs. human plan-comparison] (Fleury et al., 2025). Although this study is not directly immunoradiotherapy and is modest in size, it demonstrates the feasibility of AI for radiation-modality decision‐support in UM.

## 4 Multi-omics integration and immune landscape characterisation

Comprehensive understanding of uveal melanoma immunobiology requires integrated analysis across multiple biological layers and data modalities. AI excels at integrating such high-dimensional datasets to reveal complex interactions between tumor genetics, immune composition, and radiotherapy sensitivity determinants. In UM, characteristic mutations in GNAQ and GNA11 genes activate downstream MAPK and YAP signaling pathways, leading to immune evasion through multiple mechanisms including reduced antigen presentation and cytokine suppression (Păsărică et al., 2024; Jin et al., 2020). Simultaneously, BAP1 loss correlates strongly with a profoundly immunosuppressive microenvironment characterised by diminished cytotoxic T-cell infiltration and increased macrophage recruitment (Fu et al., 2022). AI-driven computational frameworks can systematically integrate these genomic features with transcriptomic data and advanced imaging biomarkers to classify tumours according to immune-activity levels, which substantially helps clinicians determine which patients may derive meaningful benefit from immunoradiotherapy approaches.

Advanced machine-learning approaches such as autoencoders and graph-neural networks (GNNs) have been successfully employed to uncover hidden molecular clusters within complex UM datasets (Yao et al., 2024; Hegde et al., 2025). These sophisticated models reveal immune-relevant molecular subtypes with differential expression of critical checkpoint molecules including PD-L1, CTLA-4, and LAG3 (Spiliopoulou et al., 2023; Issam et al., 2023). By integrating this molecular information with comprehensive clinical-outcome data, AI systems can identify robust biomarkers predictive of response to combined radiotherapy–immunotherapy regimens.

Moreover, single-cell RNA sequencing (scRNA-seq) data, analysed via AI-based dimensionality-reduction and clustering algorithms, provide unprecedented resolution into the cellular diversity and functional states within the UM tumour microenvironment (Mezheyeuski et al., 2023; Tang et al., 2024). These high-resolution analyses have identified exhausted CD8^+^ T-cell populations and immunosuppressive macrophage subsets as key determinants of therapeutic resistance mechanisms (Chen et al., 2024). Beyond genomic/transcriptomic dimensions, proteomics and metabolomics data further refine our understanding of UM biology. AI can process complex proteomic spectra to detect radiation-induced alterations in critical signalling networks (Jiang et al., 2023; Faião-Flores et al., 2019), such as upregulation of DNA-damage-repair pathways or oxidative-stress responses. Similarly, metabolomic profiling interpreted through AI models can uncover distinct metabolic signatures linked to radioresistance, including characteristic alterations in lipid metabolism or mitochondrial activity (Maudsley et al., 2011). Integrating these multidimensional molecular profiles with quantitative imaging biomarkers creates a comprehensive representation of tumour behaviour, enabling precise modelling of immunoradiotherapy-response patterns and resistance mechanisms.

One particularly innovative direction involves the development of “digital twins” for personalised treatment simulation and optimisation. These AI-driven virtual models integrate patient-specific omics data, imaging characteristics and clinical parameters to predict tumour evolution under different therapeutic scenarios (Stålhammar and Gill, 2023). In the context of uveal melanoma, digital twins remain largely conceptual and exploratory, and prospective clinical validation is still lacking. Nevertheless, such approaches hold potential as research tools to investigate complex immune responses and to inform future strategies for optimising immunoradiotherapy (Stålhammar and Gill, 2023). Such approaches represent the next evolution of precision medicine—transforming empirical treatment selection into truly data-driven therapy design based on individual patient characteristics.

In sum, the multi-omics-driven AI paradigm holds the promise of simultaneously addressing tumour genotype, phenotypic heterogeneity, immune contexture and radiotherapy-response determinants—thus enabling fully personalised immunoradiotherapy strategies in UM.

## 5 Current challenges and future perspectives

In contrast to existing reviews that broadly discuss artificial intelligence in oncology or immunotherapy, the present review specifically focuses on AI as an enabling technology for integrating radiotherapy-induced immune modulation with immunotherapeutic responsiveness in uveal melanoma. This disease context, characterized by immune privilege, low mutational burden, and a strong reliance on radiotherapy, provides a unique model in which AI-driven integration is not optional but essential.

Despite remarkable progress in AI applications for oncology, several significant challenges hinder the full integration of these technologies into immunoradiotherapy protocols for uveal melanoma. Most existing AI models rely on relatively small, single-center datasets, which increases the risk of overfitting and constrains generalizability, while external validation across independent cohorts remains scarce—a limitation particularly pronounced for rare tumors such as UM. In addition, imaging-derived radiomic features are indirect surrogates of tumor biology and immune activity, which may not fully capture dynamic immune responses within the tumor microenvironment. These challenges underscore the need for larger, multicenter datasets, rigorous external validation, and careful interpretation of AI-driven predictions.

One major limitation is data scarcity—UM is a rare cancer, and available imaging and multi-omics datasets remain relatively small and heterogeneous compared to more common malignancies. The development of robust, generalizable AI models requires large, high-quality, meticulously annotated datasets that adequately capture the diversity of imaging modalities, treatment protocols, and genetic backgrounds. Collaborative data-sharing initiatives and privacy-preserving federated learning frameworks may offer viable solutions by enabling distributed model training across multiple institutions without compromising patient data confidentiality. Another critical challenge lies in model interpretability and establishing clinical trust. Deep learning models often function as “black boxes,” providing predictions without transparent mechanistic explanations (Gallée et al., 2023). For oncologists to confidently adopt AI-guided recommendations in high-stakes clinical decisions, the algorithms must offer interpretable, explainable outputs that align with biological understanding and clinical reasoning. Integrating attention mechanisms, feature importance mapping, and visualization tools can help clarify how AI models generate specific predictions, thereby increasing clinical confidence and facilitating regulatory approval processes. Furthermore, the successful translation of AI-driven insights into routine clinical workflows requires sustained interdisciplinary collaboration between ophthalmologists, radiation oncologists, medical physicists, immunologists, computational biologists, and data scientists. Standardized validation protocols and prospective clinical trials will be essential to definitively demonstrate that AI-enhanced immunoradiotherapy improves patient outcomes compared with conventional approaches (Amirahmadi et al., 2023). Ethical and legal considerations, including data security protocols, algorithmic bias mitigation, and comprehensive patient consent procedures, must also be systematically addressed to ensure responsible and equitable implementation across diverse patient populations. In future, AI is expected to play an increasingly central role in precision oncology for UM. Advances in computational pathology, digital twin modeling, and real-time adaptive treatment planning may enable highly personalized immunoradiotherapy protocols tailored to individual patient dynamics. Combining AI with emerging technologies—such as molecular imaging, spatial transcriptomics, and nanodosimetry—may improve understanding of complex radiation–immune interactions at high spatial and temporal resolution. Over time, the integration of AI, immunology, and radiation oncology could transform uveal melanoma from a treatment-refractory malignancy into a model for intelligent, data-driven cancer care. These approaches may also provide insights applicable to other challenging cancers (Su et al., 2025b).

In conclusion, AI tools hold substantial potential to guide the design of future immunotherapy trials in uveal melanoma. By integrating multi-omics, imaging, and clinical data, AI can support biomarker-driven patient stratification, enabling more precise selection of individuals most likely to benefit from specific immunoradiotherapy regimens. Furthermore, AI-assisted modeling can inform adaptive trial designs, optimize dosing schedules, and assist in endpoint selection, ultimately enhancing trial efficiency and the likelihood of demonstrating clinical benefit. Incorporating these AI-driven strategies into trial planning could accelerate the translation of precision immunoradiotherapy into routine clinical practice.
